# Steroid-Refractory Autoimmune Myocarditis after Pembrolizumab Therapy: Failure of Equine Anti-Thymocyte Globulin to Prevent Heart Failure

**Published:** 2019-01-15

**Authors:** NV Baclig, C Ngo, AC Yeh, SH Chung, A Cheng, J Grim, SA Graf, KC Yang

**Affiliations:** 1 Department of Medicine, University of Washington, USA; 2 Division of Cardiology, Department of Medicine, University of Washington, USA; 3 Division of Hematology, Division of Medical Oncology, Department of Medicine, University of Washington, USA; 4 Department of Pharmacology, Veterans Affairs Puget Sound Health Care System, USA; 5 Division of Medical Oncology, Department of Medicine, University of Washington, USA

**Keywords:** Anti-thymocyte globulin, Pembrolizumab, Immune myocarditis, Immune checkpoint inhibitor, Immunotherapy, Immune related adverse effects (irAE)

## Abstract

While immune checkpoint inhibitors (ICIs) are improving outcomes for many cancers, they can have severe adverse effects. Though cardiac immune-related adverse effects (irAEs) are rare, they have considerable morbidity and mortality. Prior case studies have demonstrated successful treatment of ICI induced autoimmune myocarditis with a variety of immunosuppressive regimens. This case describes steroid-refractory autoimmune myocarditis after treatment with pembrolizumab. Treatment with equine anti-thymocyte globulin, a regimen previously documented to reverse ICI induced autoimmune myocarditis, temporarily improved clinical status and cardiac biomarkers, however eventually failed to prevent progression to heart failure and cardiovascular death. This case highlights the importance of early stress-dose steroids, identifies troponin as a potential marker of treatment response, and underscores the value of collaboration between oncology and cardiology for optimal management.

## Introduction

Immune checkpoint inhibitors are a class of monoclonal antibodies that interact with CTLA4 and PD-1 molecular pathways to potentiate the immune system. They have proven benefit in a wide range of malignancies and are typically well-tolerated; however, a fraction of patients experience irAEs with a range of severity [1]. Cardiac toxicity from ICIs, including myocarditis, atrioventricular block, and cardiomyopathy, are rare and incompletely understood [2,3]. Some reports have demonstrated reversal of these toxicities with corticosteroid treatment [4], however the majority of cases have been fatal without additional immunosuppressive agents. Nivolumab-induced myocarditis refractory to steroids has been successfully treated with equine anti-thymocyte globulin (ATG) in one reported case [5]. Here we present a case of steroid-refractory autoimmune myocarditis secondary to pembrolizumab that was treated with a similar regimen, but failed to prevent progression to heart failure and cardiovascular death.

## Case Presentation

An 80-year-old man with hypertension was diagnosed with locally advanced gastroesophageal junction adenocarcinoma in 2014 and underwent partial gastrectomy and Ivor-Lewis esophagectomy. Approximately a year later, he was diagnosed with biopsy-proven metastatic disease and underwent focal radiation to pleural metastases followed by capecitabine and then 5-fluorouracil and oxaliplatin, none of which was well tolerated. Molecular analysis of the tumor showed deficiency in the mismatch repair pathway and therefore pembrolizumab was initiated. Several days after his second infusion, he developed left eye ptosis, bilateral leg pain, and generalized weakness. He presented to the emergency department after three days of worsening symptoms, where he was found to be bradycardic with a new left bundle branch block. Additional evaluation revealed cardiac, liver, and skeletal muscle damage with elevated troponin T (>1.5ng/mL), creatinine phosphokinase, brain natriuretic peptide, and transaminases.

**Figure 1:**
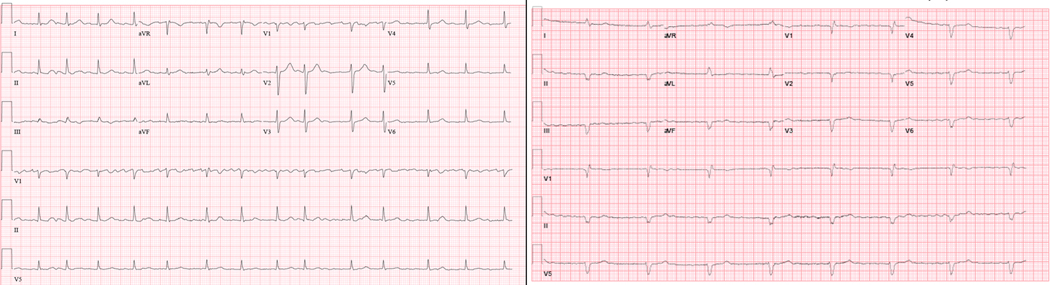
(a) Baseline ECG 3 years prior to admission. (b) Presenting ECG demonstrating atrial fibrillation with complete heart block and ventricular escape rhythm.

**Figure 2:**
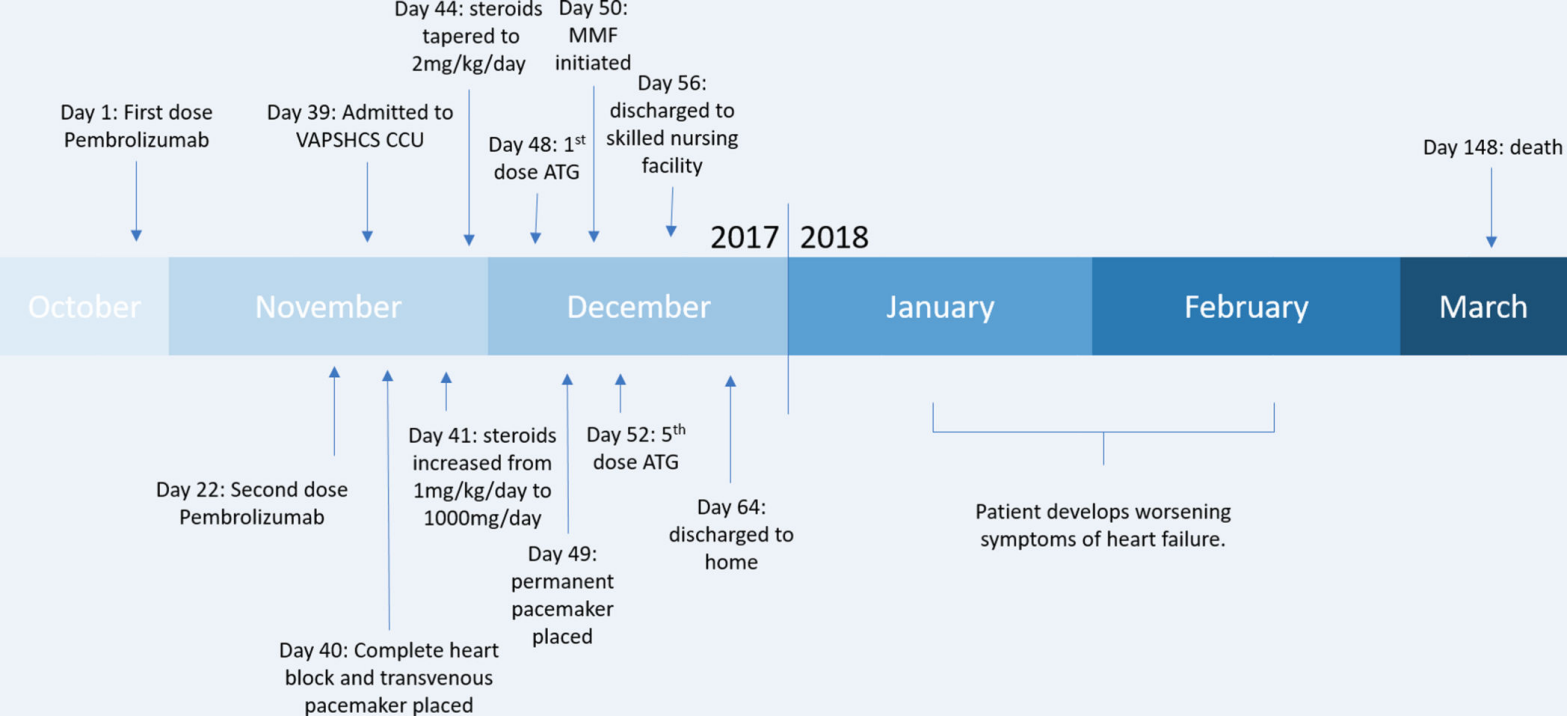
Timeline of major case events.

He was diagnosed clinically with autoimmune myocarditis related to pembrolizumab and started on methylprednisolone 1mg/kg/day. Transthoracic echocardiogram demonstrated concentric hypertrophy of the left ventricle with preserved ejection fraction. Viral PCR panel was negative for cardiotropic viruses. He quickly progressed to complete heart block (Figure 1) and a temporary intravenous cardiac pacer was placed. With limited improvement clinically, his immunosuppression was increased to 1000mg/day of methylprednisolone for three days. On this regimen, he noted symptomatic and laboratory improvement. However, when his steroids were tapered to 2mg/kg daily, both his symptoms and cardiac biomarkers worsened. He was thus initiated on an ATG regimen as described by a case of successfully-treated nivolumab-related autoimmune myocarditis [5]. He was continued on steroids and received 500mg of ATG followed by 250mg daily thereafter for a total of five days. Unlike the prior report, we were not able to titrate each dose to daily CD3 levels due to the turnaround time for the study, however the CD3 level after his first dose was < 50/uL, which was the targeted on-treatment level in the prior study [5]. On day three of ATG, he was started on mycophenolate mofetil (MMF) 1gram twice daily. While his troponin levels decreased and his symptoms improved, his complete heart block persisted. Given concerns that his immunosuppression regimen would lead to poor wound healing, he underwent permanent, Micra^®^ leadless pacemaker placement. He continued to improve clinically and was discharged to a nursing facility on 1.5mg/kg of prednisone and 1500mg MMF twice daily. Over the following 2 months, his steroids were tapered by 10mg every 3–5 days and he experienced worsening symptoms of diastolic heart failure. Transthoracic echocardiograms demonstrated elevated pulmonary artery systolic pressures, elevated right atrial pressures, and low normal systolic function. After discussions with his cardiology and oncology teams, he was transitioned to hospice and died 3 months after his initial presentation (Figure 2).

## Discussion

With increased use of immunotherapy for many cancers, a greater clinical understanding of irAEs is emerging. While the activated T-cell response that immunotherapy provokes can be effective against neoplastic cells, it is non-specific, leading to irAEs in a broad spectrum of organs. The toxicity profile of ipilimumab, the first FDA-approved drug in this class, has been well described and includes endocrinopathies, dermatitis, hepatitis and pneumonitis [6]. PD1 inhibitors have a slightly different irAE profile, namely dermatologic phenomena and gastrointestinal upset [1]. With increased use, more serious irAEs have come to light. A large review of patients with metastatic melanoma treated with nivolumab or pembrolizumab reported irAEs in nearly half of patients, with affects spanning the respiratory, musculoskeletal, nervous, and hematologic systems [7]. Cardiac irAEs in this report were rare, affecting only 1% of patients. They included relatively benign effects such as sinus tachycardia and new hypertension as well as severe cardiomyopathies and fatal myocarditis [7]. Several other case reports have described fatal outcomes from myocarditis after nivolumab use [8–10].

The prevalence of myocarditis after ICI therapy varies in the literature. An interrogation of the Bristol-Myers Squibb corporate safety databases by Johnson et al. describes severe cardiac complications as rare, with only 18 drug-related events of myocarditis among 20,594 patients (0.09%) treated with nivolumab or ipilimumab [11]. A pharmacovigilance registry at the Gustave Roussy Cancer Center in Paris suggests that these events are even more rare, after surveillance of 388 patients on ICIs for 18 months revealed only 1 cardiovascular adverse event [12–16]. However, a recent retrospective multicenter registry demonstrated a higher estimated prevalence of 1.14% [15].

Regardless of prevalence, these cardiac toxicities involve significant morbidity and mortality. A recent case-control retrospective study reported ICI-associated myocarditis to have a high rate (46%) of cardiovascular death, cardiogenic shock, cardiac arrest, and hemodynamically significant complete heart block, with a 4-fold increased risk when the troponin T was ≥ 1.5ng/ml [15]. Case reports have begun to address treatment strategies, describing variable success using high-dose corticosteroids with or without additional immunosuppressive agents such as infliximab, tacrolimus, intravenous immunoglobulin (IVIG), and MMF [2,13,15]. In a single case study of immune myocarditis secondary to nivolumab, treatment with corticosteroids and IVIG failed to produce clinical improvement, however subsequent equine ATG with MMF was shown to resolve cardiac arrhythmias, normalize cardiac enzyme elevations, and reverse complete heart block [5]. Further, this case demonstrated pathologic resolution, with pre-treatment cardiac biopsies demonstrating evidence of immune-mediated myocarditis and post-treatment biopsies demonstrating myocardial repair [5]. Subsequently, a second case has been published which reports successful reversal of cardiogenic shock secondary to combination ipilimumab and nivolumab using ATG and high-dose steroids [16–20].

**Figure 3:**
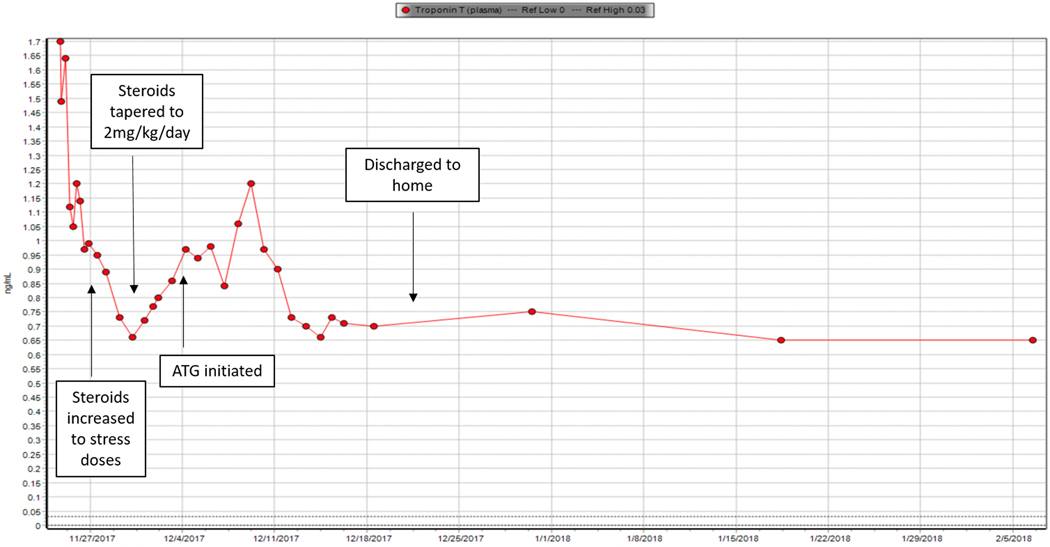
Troponin level trend from time of admission.

In the case described here, pembrolizumab was initiated for metastatic gastroesophageal junction adenocarcinoma after intolerance of prior systemic therapies soon after the drug was granted accelerated FDA approval for this indication based on KEYNOTE 059 study results [14]. The patient presented with elevated cardiac biomarkers and new heart block, which was suspicious for myocarditis. Although not confirmed with a cardiac biopsy or MRI, myocarditis was presumed given the absence of other explanations for these clinical findings such as type 1 myocardial infarction or stress-induced cardiomyopathy. As viral causes for myocarditis was ruled out, pembrolizumab-induced myocarditis was suspected, especially as his cardiac enzymes improved with immunosuppression. Unfortunately, attempts to wean steroid therapy were met with clinical worsening. Referencing the case report described above, he was treated with equine ATG followed by MMF with improvement, however, over the next several weeks, he developed symptoms and echocardiographic findings consistent with diastolic heart failure. Notably, his troponin T remained elevated > 0.6ng/mL (Figure 3), suggesting persistent myocarditis. He progressed to diuretic-refractory diastolic heart failure and, after transitioning to hospice, passed away.

This case highlights important considerations in the management of ICI-associated myocarditis. First, prompt administration of pulse-dose corticosteroids should be considered. In this case, clinical improvement was only seen when doses reached this threshold and is consistent with expert management guidelines that recommend starting with pulse-dose steroids [2]. Second, the outcome suggests that there may be patient characteristics that dictate response to immunosuppressive regimens. Further study may elucidate whether age, time to treatment, or severity of initial presentation can predict outcomes when treating with ATG. Third, while serial troponins have not been shown to predict occurrence of ICI-associated myocarditis [17], this case implicates persistent troponin elevation once myocarditis develops as a harbinger of treatment failure, suggesting that additional treatment modalities be incorporated. Finally, the importance of shared management between oncology and cardiology is emphasized in this case as is exemplified by the decision to implant a Micra^®^ leadless pacemaker to minimize issues with wound healing related to immunosuppression.

While management schema for immune-mediated myocarditis secondary to ICIs have been proposed [2,18,21], they continue to be based on early observational data. Recently, a multidisciplinary committee of stakeholders met to identify knowledge gaps and propose next steps for the development of an approach for treatment [19]. They identified what this report also highlights; that much still needs to be done to develop consensus guidelines on how to treat these life-threatening complications.
